# From the attenuation of self-boundaries to inner peace: how nonordinary states of consciousness modulates self-experience and subjective well-being

**DOI:** 10.1093/nc/niag022

**Published:** 2026-08-27

**Authors:** Pierre De Oliveira, Hélène Meunier, Audrey Breton, Michael Dambrun

**Affiliations:** Psy-DREPI (EA7458), Université Bourgogne Europe, Dijon, BP 26513-21065, France; EiSCo, Espace d’Investigation en Science Contemplative, France; Laboratoire de Neurosciences Cognitives et Adaptatives, UMR 7364, Université de Strasbourg, Strasbourg, France; Centre de Primatologie de l’Université de Strasbourg, Niederhausbergen, France; Trancescience Research Institute, 7B rue de Monceau, 75008 Paris, France; EiSCo, Espace d’Investigation en Science Contemplative, France; Université Clermont Auvergne (UCA), LAPSCO CNRS UMR 6024, LISC INRAE UR 1465, Clermont-Ferrand, France

**Keywords:** nonordinary states of consciousness, selflessness, well-being, minimal self, self-transcendence

## Abstract

Nonordinary states of consciousness induced by mind–body practices are increasingly studied for their potential to support self-transcendence and psychological well-being. According to the Self-centered/Selflessness Happiness Model (SSHM), sustained well-being may arise from a shift away from self-centered mental processes toward a more decentered, and interconnected sense of self. This study explored how a volitional altered state of consciousness (auto-induced cognitive trance—AICT) impacts different dimensions of self-experience and their relationship to well-being. Using a mixed-methods design, trained practitioners (*N* = 17) recalled two contrasting experiences: an ordinary waking state (a recent mealtime) and a self-induced trance. In each condition, they described their subjective experience, with a focus on various dimensions of self-consciousness such as self-concept, agency, bodily boundaries, self-location, and temporal awareness. Subjective well-being was also assessed using the Authentic Happiness Scale. Results showed that, compared to ordinary consciousness, the AICT state induced profound alterations in both narrative and minimal self-experience, including reduced self-referential thought, a more diffuse and decentered self-location, diminished sense of agency, attenuated bodily boundaries, and heightened feelings of oneness. These experiential changes were accompanied by increased subjective well-being, particularly in the form of inner peace. Correlational analyses yielded preliminary findings consistent with the hypothesis that these emotional benefits may be primarily associated with transformations in minimal self-related processes, though replication with larger samples is needed to confirm this pattern. These implications are discussed.

## Introduction

Understanding how and why mind–body practices impact well-being is one of the key challenges in contemplative sciences (Dambrun and Ricard 2011, Dahl *et al*. 2020, Timmermann *et al*. 2023). In the present study, we use the term “mind–body practices” to refer to structured practices that intentionally engage both bodily processes (e.g. posture, movement, interoception) and attentional or experiential regulation, typically within a socio-culturally embedded framework (e.g. meditation, yoga, or breath-based practices) (Vago and Silbersweig 2012, Farb *et al*. 2015, National Center for Complementary and Integrative Health 2021). While psychedelic substances are not considered mind–body practices in this sense, as they do not involve volitional bodily engagement or deliberate attentional training, research on psychedelic-induced states is nonetheless mobilized throughout this introduction as a phenomenologically relevant comparative framework. Psychedelics reliably produce profound alterations in self-experience that overlap with those documented in contemplative practices, and the parallels between these two literatures help to identify shared mechanisms underlying nonordinary states of consciousness and self-transformation.

Recently, an emerging body of research has suggested that these effects may be partly due to profound changes in the structures of the self (Hölzel *et al*. 2011, Vago and Silbersweig 2012, Desbordes 2019). The Self-centered/Selflessness Happiness Model (SSHM; Dambrun and Ricard 2011), for instance, posits that happiness, understood as a form of subjective well-being characterized by a stable sense of inner peace and a feeling of harmony with one’s environment rather than transient hedonic pleasure, is intimately linked to self-consciousness states such as selflessness, a psychological mode of functioning in which the individual self is experienced as a flexible and interconnected entity (Berkovich-Ohana *et al*. 2024). Recent empirical studies have supported this proposition, documenting the psychological mechanisms associated with this “selfless” state (i.e. state or trait) and how these mechanisms are related to the emergence of positive emotions, emotional stability, a sense of harmony, and well-being (Garland and Fredrickson 2019, Pellerin *et al*. 2022, Dambrun *et al*. 2024). However, it is important to note that despite this growing body of evidence regarding the psychological consequences of a selfless perspective in individuals, this research has primarily been conducted in the context of studies on the effects of meditation practice (Garland *et al*. 2015, Gu *et al*. 2015, Dahl *et al*. 2020). In other words, a range of practices where experiences of reduced self-salience are seen as a path to happiness has been proposed and discussed by various contemplative traditions (Pawar 2017) and scholars (Dambrun and Ricard 2011, Berkovich-Ohana *et al*. 2024).

In this study, we aimed to extend current contemplative research on self-related transformation and well-being by examining the subjective effects of a lesser-known body–mind practice: auto-induced cognitive trance (hereafter AICT; see Flor-Henry *et al*. 2017, Collignon *et al*. 2025, Oswald *et al*. 2023, 2025, Vanhaudenhuyse *et al*. 2024). AICT is an emerging mind–body practice that induces a nonordinary state of consciousness through self-induction techniques involving personal sounds, movements, and gestures. Recently introduced in the scientific literature, AICT has been defined as “a volitional non-ordinary state of consciousness characterized by a lucid but narrowed awareness of external surroundings” (Oswald *et al*. 2023, 2025).

The development of AICT was initially inspired by attempts to reproduce and optimize rhythmic auditory patterns used in traditional trance practices, such as Mongolian shamanic drumming. Early observations showed that exposure to specific sound loops could facilitate access to trance states in a majority of participants, leading to the progressive development of a standardized induction protocol. This protocol now relies primarily on self-generated processes, including bodily movements and vocalizations, and can ultimately be performed without external auditory support (Grégoire *et al*. 2021).

Unlike traditional or secular meditation practices that emphasize stillness and metacognitive monitoring, AICT engages practitioners in dynamic and often involuntary physical and emotional expression. It is typically characterized by rhythmic movements, spontaneous vocalizations, vivid mental imagery, and deep sensory immersion (Grégoire *et al*. 2021, Gosseries *et al*. 2024). In practice, sessions generally begin with a phase of auditory and bodily engagement, followed by a progressive increase in absorption and sensory immersion. This process often leads to spontaneous motor and emotional expressions, accompanied by reduced voluntary control and altered bodily awareness. While the precise unfolding of the experience may vary across individuals and sessions, these core elements provide a common structure.

Although AICT has been recently formalized in a scientific context, it shares certain phenomenological features with other practices involving nonordinary states of consciousness, such as trance-related or shamanic practices, particularly in its use of rhythmic stimulation, bodily engagement, and altered self-experience. However, unlike these traditional practices, in which the cultural and symbolic context plays a constitutive role in shaping the phenomenology of the experience, AICT is taught in a secular and standardized framework, without reliance on ritual, symbolic systems, or culturally specific belief structures. This does not eliminate socio-cognitive influences entirely, as practitioners share a common training framework and may hold expectations about the nature of the experience, but it allows for a more controlled investigation of the intrinsic phenomenological features of trance-like states. Because of its embodied and expressive nature, AICT offers a novel and distinct phenomenological entry into altered states of consciousness, providing a valuable context for investigating how the structures of the self, particularly the bodily, spatial, and agentive dimensions, may be modulated, diminished, or transformed. Furthermore, following initial training, practitioners develop the capacity to self-induce trance states autonomously, in varied personal contexts and without external support, which means that sessions may differ considerably across individuals in terms of duration and setting while retaining these core phenomenological features.

For over a century, several theoretical frameworks have aimed to map out the various processes underlying individuals’ sense of self. Among the proposals made, it is now widely accepted that the sense of self is a complex notion that should be considered as a multidimensional construct, including several extended processes such as somatosensory, experiential, agentive, narrative, social, and cultural components (Gallagher 2000, Millière *et al*. 2018, Gallagher *et al*. 2024). To facilitate its operationalization, several researchers have proposed distinguishing primarily two major components of the self: the “narrative self” and the “minimal self” (Gallagher 2000). These two components of the self are related to two types of processes linked to distinct brain regions and networks (Christoff *et al*. 2011).

Thus, the “narrative self” is made up of the stories we tell ourselves about who we are. This component of the self is characterized by a set of mental activities, including self-thoughts, autobiographical memories, future projections, and identity characteristics. The “narrative self” provides the individual with a coherent image of the self over time, with a past, present, and future, and leads to the feeling of being the one who thinks the thoughts or the one who experiences the emotions (e.g. “I am a confident person,” “I am a caring parent,” or “I am someone who tends to worry a lot”). According to this distinction, the “minimal self” refers to the self as “I,” an immediate form of self-awareness anchored in the body and its sensations that does not require explicit reflection or narration. It is a pre-reflective sense of being the subject of one’s experience, without an extension in time. It involves self-referential sensorimotor experiences, such as the sense of agency (i.e. the feeling that I am the initiator or source of action, movement, or thought) and the sense of ownership (i.e. the feeling that my body belongs to me and that I am the subject of the experience). The minimal self also largely relies on bodily processes such as proprioception, which is the ability to perceive the position and movements of one’s own body.

While this distinction between minimal and narrative self has proven highly influential, recent work in embodied cognition suggests that it should not be interpreted as implying a strict separation between bodily and narrative dimensions of selfhood. In particular, the narrative self is increasingly understood as grounded in embodied processes and shaped by the individual’s history of sensorimotor engagement with the world (Menary 2008, Køster 2017, Dings 2019). From this perspective, self-narratives are not solely the product of abstract or reflective mental activity but are constrained and informed by pre-reflective embodied patterns, including habitual ways of perceiving, acting, and engaging with the environment (Miyahara and Tanaka 2025). Embodiment and narrativity are therefore deeply intertwined: alterations in bodily experience may not remain confined to the minimal self but may propagate upward to reshape narrative identity itself. This interdependence is particularly relevant in the context of AICT, which engages practitioners through dynamic sensorimotor processes. If AICT induces profound modifications of minimal self-experience, such as altered agency, bodily boundaries, and self-location, these changes may not leave the narrative self untouched but may simultaneously reconfigure the experiential ground on which narrative self-processes rest.

### Mind–body practices and the narrative self

Although empirical research on AICT remains limited, a few recent studies have begun to document its phenomenology (Vanhaudenhuyse *et al*. 2024, Collignon *et al*. 2025, Oswald *et al*. 2025). These studies suggest that AICT involves altered body perception, changes in the sense of self, vivid imagery, absorption, and difficulties describing ongoing experience. However, this literature remains relatively broad in scope and was not specifically designed to examine narrative self-processes, such as autobiographical self-representation or self-referential thinking.

In this context, research on meditation and psychedelics provides the most relevant comparative framework currently available for generating hypotheses about narrative self-processes in AICT. These contexts share with AICT core phenomenological features, including profound alterations in absorption, bodily awareness, and self-referential processing, as well as a common shift away from ordinary self-centered modes of experience. The retrospective recall methodology developed by Droit-Volet and Dambrun (2019) with experienced meditators is also directly applicable here, as AICT practitioners similarly develop the capacity to access and recall distinct nonordinary states of consciousness.

Consistent with this rationale, a growing body of research demonstrates that various contemplative and altered state-inducing approaches, including meditation and psychedelic states, can profoundly alter the narrative dimension of self-consciousness (Hölzel *et al.* 2011, Berkovich-Ohana and Glicksohn 2014, Britton *et al.* 2021). Practices such as focused attention or open monitoring mindfulness have been shown to reduce self-referential mental activity, including rumination and mind wandering (Britton *et al.* 2021, Mrazek *et al*. 2012). Programs like Mindfulness-Based Stress Reduction (MBSR) and Mindfulness-Based Cognitive Therapy (MBCT) also decrease maladaptive self-focused thought patterns (Gu *et al*. 2015, Perestelo-Perez et *al*. 2017). As suggested in many contemplative traditions, this attenuation of narrative self-related processes is thought to stem from a decentered mode of awareness, an ability to observe thoughts as transient mental events without identification (Bernstein *et al*. 2015). This shift allows for greater psychological distance, reduces the grip of self-referential content on emotion, motivation, and behavior, and fosters a more fluid self-representation. Interestingly, similar effects are observed in psychedelic states. Studies report that substances like psilocybin or lysergic acid diethylamide (LSD) lead to temporary ego dissolution, a marked decrease in self-referential thoughts, and disruptions in autobiographical memory (Nour *et al*. 2016, Millière *et al*. 2018, Stoliker *et al*. 2022). These states are often accompanied by decreased activity in the Default Mode Network (DMN), a neural hub associated with narrative self-processing and enhanced absorption in the present moment (Nour *et al*. 2016). In addition to these altered states, profound emotional experiences such as awe have been shown to impact how individuals define themselves. In such experiences, individuals tend to shift away from self-centered concerns and describe themselves in broader terms (Shiota *et al*. 2007). This more interconnected sense of self has also been observed in meditation studies. Dambrun and Ricard (2011) describe a selflessness-based mode of functioning in which the individual feels embedded within, rather than separate from, the surrounding world. De Oliveira *et al*. (2024) found for instance that in experienced meditators, an increased ability to observe internal states without identifying with them predicted a more interconnected self-identity, less bounded by rigid internal/external distinctions and more attuned to a broader continuity with others, nature, and even past and future generations.

It’s important to note that some contemplative experiences can also affect the narrative self in a more disruptive way. Droit-Volet and Dambrun (2019) revealed, for example, a narrowing of access to the self-concept during deep meditative states among expert meditators. When asked how they perceive themselves as individuals during meditation, they report a sensation of their personal identity disappearing and a difficulty in describing themselves. Intense absorption in a meditative state seems to diminish access to autobiographical memory for self-definition (see also Ataria *et al*. 2015). Millière (2022) report similar testimonies from psychedelic users. Many “trip reports” indicate a temporary absence of self-related thoughts and a loss of access to autobiographical memory information (see also Metzinger 2024).

### Mind–body practices and minimal self

As with the narrative self, empirical work specifically examining minimal self-processes in AICT remains scarce. Existing studies suggest that AICT involves alterations in bodily awareness, sense of self, and sensorimotor experience (Vanhaudenhuyse *et al*. 2024, Collignon *et al*. 2025), but these aspects have not been systematically investigated using established frameworks of minimal selfhood. For this reason, and consistent with the rationale developed in the preceding section, we draw on research from meditation and related altered state contexts, including psychedelic research, where minimal self-processes have been more extensively studied. Although these approaches differ from AICT in their mode of induction, they share key phenomenological features, including altered embodiment, reduced sense of agency, changes in bodily boundaries, and shifts in self-location, that make them directly relevant for generating hypotheses about minimal self-transformations in AICT.

As proposed by Berkovich-Ohana and Glicksohn (2014), the attenuation of narrative self-related processes is closely associated with profound modifications in components of the minimal self. As with the narrative self, most of these observations originate from research on meditation, and to a lesser extent, from psychedelic or virtual reality studies (Glowacki *et al*. 2022, Glowacki 2024). For instance, several qualitative and neurophenomenological investigations have explored how the minimal self is altered during deep meditation, particularly in experienced practitioners from Satipatthana and Theravada Vipassana traditions. For example, Dor-Ziderman *et al*. (2013) instructed 12 long-term mindfulness practitioners to voluntarily induce a “selfless” mode of awareness, asking them to try to experience what is happening in the present moment when they are not in the center. Several participants reported a diminished or absent sense of ownership, illustrated by statements such as: “I understood that it was just a sensation, it was not the hand itself... there was a deep thought that all this was not mine” or “There was an experience but it had no address, it was not attached to a center or subject” (p. 6). Similar findings were reported by Droit-Volet and Dambrun (2019), who found that during recollection of deep meditative states, practitioners frequently expressed a weakened feeling of ownership: “Very low feeling of being this body”; “My feeling of being this body is pretty weak” (p. 58; see also Ataria *et al*. 2015). This diminished sense of bodily ownership was sometimes accompanied by a loss of agency. Participants described the absence of voluntary control over bodily actions, with movements unfolding independently of conscious intention: “There’s no more head,” “The body lives its own life,” or “The body moves alone” (Droit-Volet and Dambrun 2019, p. 58). As noted by Lindahl and Britton (2019), this loss of agency can manifest in spontaneous postures or the feeling that another force is acting through the body. These changes also extend to alterations in proprioception and bodily boundaries. Berkovich-Ohana et *al*. (2013) revealed that during meditation instructions for “spacelessness” or “timelessness,” practitioners frequently reported a disappearance or expansion of their sense of the body. Examples include: “The body was wider and wider. The body as a physical image was absent. There was a sense of open space without the bodily dimension”; “Bodily sensations were wider. Bodily space was larger” (pp. 4–6). Similarly, during “timelessness” instructions, participants described: “There was little awareness of different body parts. Little body boundaries compared to the usual feeling”; “There was a vanishing of bodily sensations. The body was not present” (Ataria et *al*. 2015, Nave et *al*. 2021). Importantly, such effects are not exclusive to expert practitioners; even novices report altered bodily boundaries after simple practices like a short body scan (Dambrun 2016, Dambrun et *al*. 2019).

Finally, reduced embodiment is often linked with altered self-location in time and space. According to Alsmith and Longo (2014), people typically localize themselves in conventional bodily areas such as the head or upper torso, which define both the perceptual origin point (egocentric frame) and the subjective center of experience. However, several studies show that this localization can shift. For example, during deep meditation, participants often struggle to determine “where they are,” reporting: “I don’t know. There is no longer any body, so the location is complicated” (Droit-Volet and Dambrun 2019, p. 59) or “There is really no address... I’m not there basically ... so there’s no real location” (Ataria et *al*. 2015, p. 141).

### Mind–body practices, sense of self change, and well-being

The potentially beneficial or harmful consequences of these self-consciousness states during mind–body practices are the subject of increasing investigation (Garland and Fredrickson 2019, Lindahl *et al*. 2019, Garland *et al*. 2022). According to the teachings of spiritual traditions, some contemplative science frameworks postulate that positive emotions and well-being arising from these practices are closely linked to the ability to move beyond a self-centered perspective (e.g. excessive self-focus, hedonic preoccupations) toward a broader sense of connection and unity (i.e. a self-transcendence view) (Dambrun and Ricard 2011, Vago and Silbersweig 2012), although these outcomes are not universal and may, in some cases, be accompanied by challenging or destabilizing experiences (Lindahl *et al*. 2019).

From an empirical perspective, there is some asymmetry in the literature regarding the respective roles of changes in the narrative self and minimal self in explaining the well-being and optimal experiences felt when the selfless mode becomes predominant. Indeed, the transition to a selfless state has mostly been studied in terms of its effects on self-related conceptual processes (Desbordes 2019, Britton *et al.* 2021). Numerous studies have shown that meditation practice or the use of psychedelics have a positive impact on mental health and well-being because they deeply affect maladaptive schemas of the narrative self (Gu *et al*. 2015, Davis *et al*. 2020). In other words, by offering greater psychological flexibility and allowing for the development of a stronger capacity for self-decentering, these practices significantly reduce self-negative evaluations such as anxiety, dysfunctional attitudes, or depressive-related thoughts (Britton *et al.* 2021, Sloshower *et al*. 2024) and also lead to feelings of greater harmony, more positive emotions, and well-being (Dambrun 2017, De Oliveira et *al*. 2024). It is important to note here that there is a close link between the narrative self and body awareness during these practices. Research has shown that returning to the present moment through body awareness is one of the mechanisms that facilitates the reduction of the narrative self’s influence on usual psychological functioning. Recently, Dambrun et *al*. (2023) demonstrated that among novices practicing a body scan, increased happiness was mediated by a change in self-location (i.e. an increase in self-location in the abdomen, torso, and peripheral limbs). This shift in self-location was associated with a decrease in the number of thoughts and concerns related to the self. The attenuation of embodied senses of self during intensive practices (e.g. loss of agency, fewer boundaries between self and world) is also associated with psychological mechanisms that may promote well-being and positive emotions (Hölzel *et al*. 2011, Dor-Ziderman *et al*. 2013). Dambrun *et al*. (2019) found that a positive emotional state, characterized by the presence of happiness and the absence of anxiety, emerges when the perceived boundary between the body and the environment becomes less perceptible during a body scan practice. The explanation provided by these authors is that when the self/not-self distinction becomes less marked, and the self is perceived as an interconnected, transient event (i.e. selflessness mode), individuals are less motivated by the need to ensure their own preservation. The hedonic principle becomes less important, and the associated anxiety is reduced. In other words, when the individual’s perceived body boundaries extend beyond the body and encompass everything that surrounds them (i.e. oneness), it is assumed that this state is accompanied by a feeling of harmony (Dambrun and Ricard 2011). These observations align with findings on the beneficial effects of mystical states and/or self-transcendence (Yaden *et al*. 2017). Generally characterized by both a decrease in self-salience and an increase in feelings of oneness, these states of consciousness are frequently associated with an increase in psychological well-being, blissful states, and self-transcendence emotions (Dambrun *et al*. 2019, Hanley *et al*. 2020, 2023, Hanley and Garland 2022).

On the other hand, adverse effects such as fear, terror, or difficult somatic experiences during intense meditative states, awe, or psychedelic experiences have been less well studied in the literature. An exception in this field is the work of Lindahl and Britton (Lindahl *et al*. 2019), who examined the challenging experiences of meditators from various traditions. Among the difficult experiences identified, practitioners reported that these changes in the self could be associated with distress, a sense of rupture, and the perception of impairment in their habitual functioning. For instance, experiencing dissociation, where the individual feels a profound sense of depersonalization or derealization (i.e. the “annihilational” component of self-transcendence experience), could be associated with the fear of developing a psychopathological disorder.

### The present study

Building on the theoretical framework outlined above, the present study pursued two main objectives: (i) to investigate changes in both narrative and minimal self-experience during AICT compared to an ordinary waking state and (ii) to examine whether these changes are associated with subjective well-being, in line with the Self-centered/Selflessness Happiness Model (SSHM; Dambrun and Ricard 2011).

Drawing on the SSHM and on converging evidence from meditation and psychedelic research, we formulated the following hypotheses:

*H1 — Narrative self-alterations.* AICT will be associated with significant alterations in narrative self-processes compared to an ordinary waking state. Specifically, participants are expected to report reduced self-referential thinking and mind-wandering (past- and future-oriented), altered temporal experience, and decreased narrative sense of agency.

*H2 — Minimal self-alterations.* AICT will be associated with significant alterations in minimal self-experience. Specifically, participants are expected to report a reduced sense of motor agency, more permeable or absent bodily boundaries, a more diffuse and decentered self-location, a shift away from an egocentric toward a more allocentric or boundaryless spatial perspective, and heightened feelings of oneness with their surroundings.

*H3 — Association with subjective well-being.* The self-related changes described in H1 and H2 will be positively associated with subjective well-being during AICT, particularly with the inner peace dimension of authentic-durable happiness (Dambrun *et al*. 2012).

To test these hypotheses, and in line with prior work on meditation-induced altered states (e.g. Droit-Volet and Dambrun 2019), we adopted a mixed-methods design combining open-ended phenomenological questions and quantitative ratings across two contrasting conditions: an ordinary waking state (recall of a recent meal) and an AICT experience.

## Method

### Participants

Seventeen participants were interviewed for this study (13 women and 4 men; *M*_age_ = 49, *SD* = 11.45). All participants had previously undergone initial training in AICT, proposed by the TranceScience Research Institute (trancescience.org). This training typically includes (i) exposure to rhythmic auditory stimulation designed to facilitate trance induction, (ii) guided practice involving bodily movements and vocalizations, and (iii) progressive development toward autonomous self-induction without external support. As such, participants likely shared a common conceptual and experiential framework regarding AICT. This shared framework encompasses not only technical knowledge of the induction protocol but also a common set of representations about the nature and expected effects of the trance state, including beliefs about its safety, its phenomenological characteristics, and its potential benefits. These shared expectations may have shaped participants’ experiences during AICT, and should be considered when interpreting the present findings.

The time elapsed between their initial training and the interview varied from a few days to several months (*M* = 15 months, *SD* = 6.40). Since their training, participants reported practicing AICT several times a week or daily (*M* = 4.29, *SD* = 1.57; i.e. “Since the training, have you practiced cognitive trance?; Item rated on a 7-point Likert scale ranging from 1 “Never” to 7 “Several times a day”) and estimated that each session lasted ~10 min on average (*SD* = 1.09). In terms of perceived intensity, participants reported that the sessions were generally moderate to fairly intense (*M* = 3.81, *SD* = .98; i.e. “In general, over the past three months, how intense have your trance experiences been?”; 5-point Likert scale ranging from 1 “Not intense” to 5 “Very intense”). Twelve participants were French nationals, two had dual nationality, one participant was Belgian, and another was Italian.

#### Ethical considerations

Participants were informed of their right to refuse or withdraw from the study at any time without facing any retaliatory action, in accordance with the Declaration of Helsinki. Consent was obtained from all respondents before participation. Participants were informed prior to the interview that it would be audio-recorded and subsequently anonymized using a research code. Inclusion criteria required fluency in French, the ability to speak, read, and understand French, and a minimum age of 18. The study adhered to all ethical guidelines outlined in the Declaration of Helsinki, and ethical approval was granted by the Ethics Committee for Research at the University of Bourgogne Franche-Comté (CERUBFC-2022-05-24-020)

#### Procedure

Upon confirmation of consent, participants were asked to provide socio-demographic information (e.g. age, gender, education level) and to indicate the extent to which they had continued practicing AICT in their daily lives since their training (i.e. frequency, duration, intensity). The interview followed the same structure as the protocol developed by Droit-Volet and Dambrun (2019) in their study on expert meditators, involving two separate phases during which participants were invited to recall and describe their subjective experience of the self during contrasting mental states (i.e. “AICT” vs. “ordinary state of consciousness”). The order of the two phases was randomized across participants.

This recall-based approach rests on the well-established capacity of episodic memory to reinstate not only the factual content of a past experience but also its associated sensory, emotional, and phenomenological dimensions (Conway and Pleydell-Pearce 2000, Tulving 2002). By inviting participants to mentally re-enter a prior experience, this method allows access to the subjective texture of states that cannot be directly observed or induced in a laboratory setting, making it particularly well suited to the study of altered states of consciousness. This approach has been successfully used in prior phenomenological and contemplative research to capture the experiential characteristics of deep meditative states in experienced practitioners (Petitmengin 2006, Droit-Volet and Dambrun 2019), and its use here follows the same rationale: practitioners who have repeatedly experienced AICT develop a detailed phenomenological familiarity with the state, which supports accurate and nuanced retrospective description.

In the “ordinary state of consciousness” phase, participants were asked to think about a moment from everyday life within a specified time frame. For this, they were instructed to recall the last time they had a meal. To help them reconnect with this moment, they were encouraged to take a few moments to naturally bring back memories of this event, such as where they were, who they were with, and what they ate and drank. In the “AICT” phase, participants were asked to recall a deep cognitive trance state they had experienced since their training. Participants were free to select any trance experience of their choice, which was not necessarily limited to the training context and could have occurred in a variety of personal settings following training. To assist them, they were invited to reconnect with the sensations, perceptions, and subjective experiences associated with this nonordinary state of consciousness. For each of these two phases, once participants indicated that they were ready, they were asked to describe what they had experienced.

### Measures

The measures were organized according to the main theoretical dimensions of interest: (i) narrative self, (ii) minimal self, and (iii) subjective well-being. Within each dimension, we combined open-ended questions and quantitative items when appropriate, in order to capture both qualitative and quantitative aspects of participants’ experience (see Appendix I in Supplemental Materials).

#### Narrative self

In this study, the narrative self was assessed through four complementary components: self-concept, mind-wandering, narrative agency, and temporal experience. For each component, we used open-ended questions and/or quantitative items.

##### Self-concept

Self-concept was explored through an open-ended question inviting participants to reflect on how they perceived themselves as individuals during the experience (e.g. “How do you perceive yourself as an individual? How would you describe yourself?”).

##### Mind-wandering

Mind-wandering was assessed both qualitatively and quantitatively (see Droit-Volet and Dambrun 2019). Qualitatively, participants were asked to describe what their mind was occupied with during the experience (e.g. “During this event, what was your mind occupied with?”). Quantitatively, they indicated, on three separate scales ranging from 0% to 100% of the time, the extent to which their thoughts were focused on the past (Q1: “During this event, what percentage of the time were your thoughts focused on the past?”), the present (Q2: “...on the present?”), or the future (Q3: “...on the future?”).

##### Narrative agency

Narrative agency was assessed through two quantitative items. The first item measured the sense of being an agent of one’s thoughts and emotions (Q4: “During this event, regarding your thoughts and emotions, do you feel like you are the agent of what you think and feel?”; 0% = “Not at all the agent,” 100% = “Completely the agent”). The second measured the sense of being an observer (Q5: “...do you feel like an observer of what you think and feel?”; 0% = “Not at all an observer,” 100% = “Completely an observer”).

##### Temporal experience

Temporal experience was assessed both qualitatively and quantitatively. Qualitatively, participants were asked to describe their experience of time during the event (e.g. “What is your experience of time during this event?”). Quantitatively, participants rated the perceived speed of time (Q6: “How fast does time go?”; 0 = “Very slowly,” 100 = “Very quickly”) and the presence or absence of time in their mind (Q7: “How present or absent is time in your mind?”; 0 = “Completely absent,” 100 = “Completely present”).

#### Minimal self

Changes in the perceived dimensions related to the minimal self were investigated through four components: self-location, perspective, sense of agency, and bodily boundaries/oneness. For each component, we used open-ended questions and/or quantitative items.

##### Self-location and embodiment

Self-location was first explored through open-ended questions asking participants where they felt their “self” was located during the experience (e.g. “Where are you located during this event? If ‘body,’ where exactly? Head, torso, elsewhere? If ‘not body,’ where exactly?”). To complement this qualitative assessment, participants rated their degree of bodily identification (Q8: “To what degree would you say you are this body?”; 0 = “Not at all,” 100 = “Completely”).

##### Perspective (egocentric vs. allocentric)

The perspective from which participants experienced the scene was assessed using quantitative items. Participants rated the extent to which they felt at the center of the scene (Q9: “Do you feel as though you are at the center of the scene?”; 0 = “Not at all,” 100 = “Completely”) and the diffuseness of their self-location (Q10: 0 = “Completely diffuse,” 100 = “Completely located at a specific point”).

##### Sense of agency

The sense of agency was assessed both qualitatively and quantitatively. Participants were asked to describe their subjective experience of control over their body and movements (e.g. “What is your sensation of control over your body and movements? Can you describe it to me?”). They also quantified this experience (Q11: scale from 0 = “No control at all” to 100 = “Complete control”).

##### Bodily boundaries and oneness

As part of the broader phenomenology associated with self-transcendence experiences (see Dambrun *et al*. 2019), the perception of bodily boundaries and self–world distinction was assessed through both open-ended and quantitative measures. Participants were first asked to describe their perception of their bodily boundaries during the experience. They then rating the clarity of their boundaries (Q12: 0 = “Completely absent boundary,” 100 = “Very distinct boundary”), the degree of perceived duality (Q13: “Do you feel there is a duality between you and the rest?”; 0 = “No duality at all,” 100 = “Complete duality”), and oneness (Q14: “Do you feel as though you are one with everything around you?”; 0 = “Not at all,” 100 = “Completely”).

#### Subjective happiness

Subjective happiness was assessed using the Subjective Authentic–Durable Happiness Scale (SA-DHS; Dambrun et *al*. 2012), administered at the end of each interview phase to capture retrospective well-being associated with each condition. The SA-DHS comprises 16 items, including 13 positively valenced items (e.g. happiness, bliss, overall well-being, serenity, plenitude) assessing authentic durable happiness, and 3 negatively valenced items (e.g. unhappiness, displeasure) included to control for compliance bias. Participants rated each item on a scale ranging from 0% (“Not at all”) to 100% (“Totally”). A composite score of subjective state happiness was calculated by averaging the 13 positively valenced items. In addition, two mean subscores were computed to reflect the dimensions identified by Dambrun *et al*. (2012): (i) Contentment (e.g. overall well-being, happiness, pleasure, bliss, satisfaction, beatitude, fulfillment, joy) and (ii) Inner peace (e.g. peace of mind, serenity, inner peace, inner calm/tranquility, plenitude).

For all measures, higher scores indicate greater levels of state happiness. These scales demonstrated satisfactory reliability, with Cronbach’s alpha values around .95.

### Data analysis

Quantitative data were analyzed using repeated-measures ANOVAs to compare AICT and ordinary states across self-related and well-being measures. Effect sizes are reported using partial eta squared (*η*^2^_p_). Repeated-measures ANOVAs were preferred over mixed-effects models given the small, fully balanced sample (*N* = 17, no missing data), which precludes reliable estimation of random-effects structures. The within-subject design inherently controls for individual differences. Nonparametric Wilcoxon signed-rank tests were conducted as robustness checks and yielded consistent results; for conciseness, only parametric results are reported (see Table 1).

**Table 1:** Descriptive and inferential statistics (means, standard deviations, *F* values, and partial eta squared) for all quantitative variables across conditions

	AICT	Ordinary	*F*	*η* ^2^ _p_
	M (*SD*)	M (*SD*)		
**Narrative self**				
Mind-wandering past (Q1)	2.06 (1.3)	13.82 (4.6)	5.71^*^	.26
Mind-wandering future (Q2)	2.65 (1.4)	31.47 (5.7)	31.9^***^	.67
Mind-wandering present (Q3)	37.03 (10)	63.53 (5.3)	8.24^*^	.34
Narrative agency—agent (Q4)	12.10 (3.3)	67.40 (6)	70.33^***^	.81
Narrative agency—observer (Q5)	54.71 (9.6)	35.29 (8.1)	2.48	.13
Time perception (Q7)	7.00 (2.9)	64.30 (6.9)	47.96^***^	.75
**Minimal self**				
Embodiment (Q8)	64.10 (9.3)	73.10 (4.3)	.67	.04
Self-location—center (Q9)	73.30 (10.5)	79.30 (7.0)	.22	.02
Diffuse vs localized self (Q10)	50.40 (10.5)	85.70 (4.2)	8.48^*^	.39
Sense of agency—body (Q11)	10.40 (3.3)	83.80 (4.1)	201^***^	.93
Bodily boundaries (Q12)	36.50 (7.2)	83.80 (5.9)	36.3^***^	.69
Self-world duality (Q13)	26.30 (8.6)	81.00 (6.7)	23.6^***^	.61
Oneness (Q14)	75.90 (8.4)	28.40 (7.4)	15.6^***^	.51
**Subjective happiness**				
Global score	74.20 (6.6)	50.5 (6.4)	6.93^*^	.30
Contentment	73.70 (7.1)	56.60 (6.8)	3.91	.20
Inner peace	74.70 (6.8)	44.30 (6.4)	8.99^**^	.36

*Note.*  ^*^P < .05, ^**^P < .01, ^***^P < .001. Q6 (perceived speed of time) could not be submitted to statistical analysis due to missing data: 11 out of 17 participants reported being unable to quantify the speed of time during the AICT experience. Time perception reported in the table therefore corresponds to Q7 (presence or absence of time in mind). The labeling system Q1 through Q14 applies to items developed specifically for this study; subjective well-being was assessed using the validated SA-DHS (Dambrun et *al*. 2012) and follows a separate presentation format.

To examine associations between self-related variables and subjective happiness, Spearman’s rank-order correlations were used, given the small sample size (*N* = 17) and potential non-normality of distributions. While repeated-measures ANOVAs are generally robust to moderate violations of normality in within-subject designs (Schmider *et al*. 2010), as further confirmed by the Wilcoxon robustness checks, correlation coefficients are more sensitive to distributional assumptions and outliers in small samples. A nonparametric approach was therefore preferred for the correlational analyses as a more conservative choice given these constraints (Bishara and Hittner 2012).

Qualitative data from open-ended questions were not subjected to formal coding or inter-rater reliability procedures. Instead, verbatim excerpts were selected to illustrate experiential patterns appearing consistently across participants’ accounts, following a phenomenological approach (Petitmengin 2006, Droit-Volet and Dambrun 2019). The thematic labels used to organize these excerpts were derived deductively from the theoretical framework guiding the study, specifically the distinction between narrative and minimal self dimensions and their established components in the literature. These labels were discussed and agreed upon among the authors to ensure their conceptual alignment with the theoretical constructs of interest. This approach does not involve systematic inductive coding or blind rating procedures, and the qualitative data are therefore presented as descriptive illustration of the quantitative findings rather than as independent evidence.

Given the exploratory nature of this study and the absence of prior data on the effects of AICT on self-related processes, no a priori power analysis was conducted, and the present study should be considered a pilot investigation. While the sample is adequately powered to detect large effects (*η*^2^_p_ ranging from .26 to .93), the risk of Type I errors cannot be excluded given the number of comparisons, and nonsignificant results, particularly in correlational analyses, may reflect insufficient power rather than a true absence of association (Type II error). Replication with larger samples is therefore necessary.

## Results

**Figure 1:**
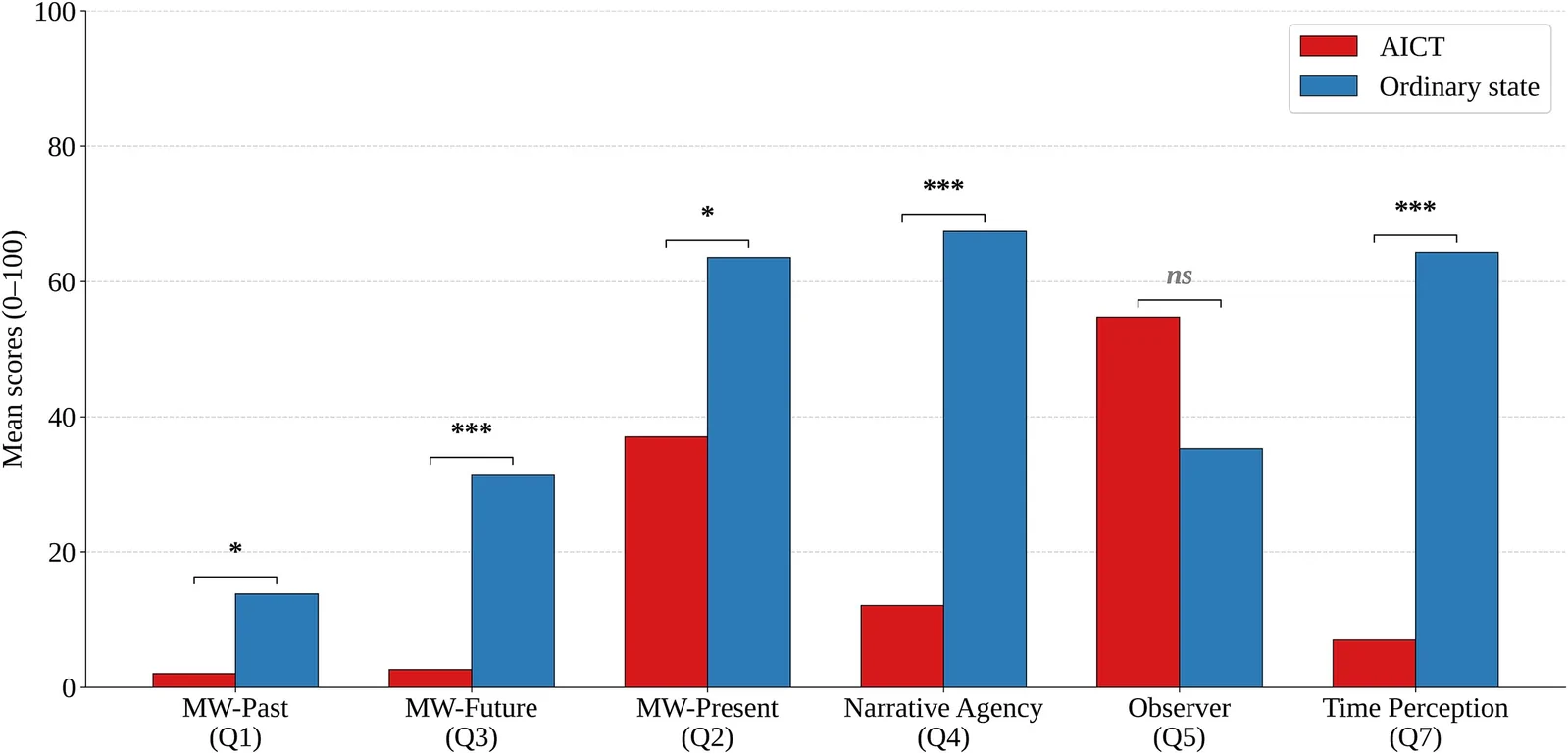
Alterations of narrative self dimensions across conditions (AICT vs. ordinary state). *Note.* MW = mind wandering. Q1–Q7 refer to the quantitative items described in the measures section. ^*^P < .05, ^***^P < .001, ns = nonsignificant.

**Figure 2:**
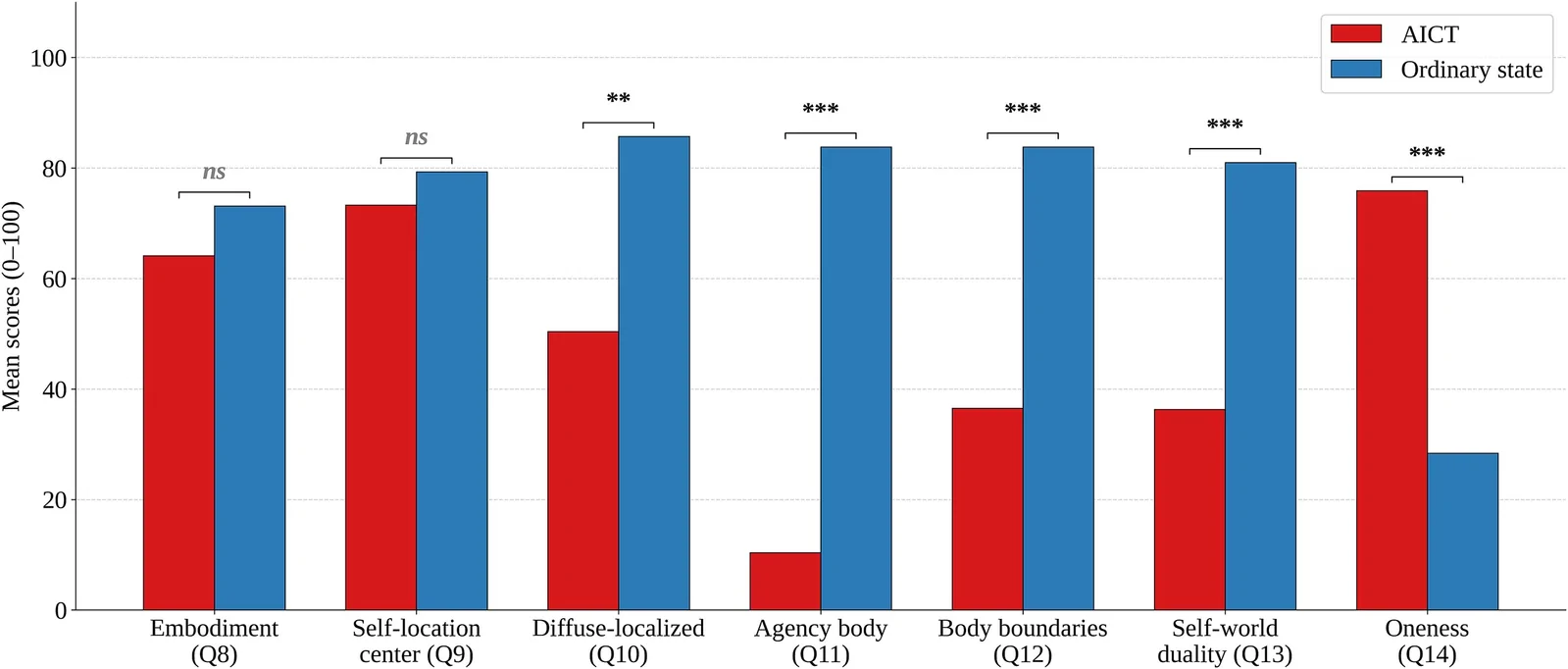
Alterations of minimal self dimensions across conditions (AICT vs. ordinary state). *Note*. Q8–Q14 refer to the quantitative items described in the measures section. ^**^P < .01, ^***^P < .001, ns = nonsignificant.

Descriptive statistics for all quantitative variables are presented in Table 1, while Figs 1 and 2 provide a visual summary of the main patterns across conditions.

### Alterations of the narrative self

Figure 1 highlights the quantitative results for each specific alteration of the narrative self.

#### Self-concept/personality

As expected, when participants were asked to describe themselves during a moment of ordinary consciousness (e.g. preparing a meal), they primarily described themselves in terms of personality traits (e.g. “Calm, responsible, maternal, and at the same time annoyed, efficient, soothing” (S.16); “I’m rather someone who is calm, attentive, and quite empathetic” (S.06); “I am as I am, meaning scattered but generous” (S.03). In contrast, during the cognitive trance state, participants’ reports suggest a disrupted access to their habitual narrative self. Several participants expressed a difficulty or inability to describe themselves (e.g. “I can’t do it” (S.08); “I don’t describe myself, I don’t know, I couldn’t describe myself” (S.12)). Others reported a sensation of being a more expansive and less distinct individual (e.g. “I feel like my bodily boundary has exploded, as if I’m invading a space—even though the people I visualize are not part of me and are somewhere else. I feel like I’m in a much larger space than my physical body” (S.9); “I feel like I’m no longer an individual, just the emotion. It’s as if the emotion takes over and I’m no longer there. I’m in a scene, witnessing it, in images. I see myself, but I don’t feel like I’m an individual” (S.06); “When I perceive myself, I feel transparent. As an individual, I don’t feel like my presence has strong significance” (S.11)) (see Table 2).

**Table 2:** Illustrative quotes of self-concept and personality trait alterations during AICT versus ordinary/habitual consciousness

	Quotes—self-concept/personality (ID)	Synthesis	Occurrence (%)
Ordinary/habitual state	“Calm, responsible, maternal, and at the same time annoyed, efficient, soothing.” (S.16) “I’m rather someone who is calm, attentive, and quite empathetic.” (S.06) “I am as I am, meaning scattered but generous.” (S.03) “I’m [first name], I’m 24 years old, I feel physically, mentally, and emotionally great.” (S.07) “I can tell you I’m very cerebral. I have a very strong analytical mind. I’m extremely rigorous.” (S.08)	Self-concept structured, stable, and reflexive.	100
AICT	“I can’t do it.” (S.08) “I don’t describe myself, I don’t know, I couldn’t describe myself.” (S.12)	A reduced access to self-description	11.8
	“I feel like my bodily boundary has exploded, as if I’m invading a space—even though the people I visualize are not part of me and are somewhere else. I feel like I’m in a much larger space than my physical body.” (S.09) “I feel like I’m no longer an individual, just the emotion. It’s as if the emotion takes over and I’m no longer there. I’m in a scene, witnessing it, in images. I see myself, but I don’t feel like I’m an individual.” (S.06) “When I perceive myself, I feel transparent. As an individual, I don’t feel like my presence has strong significance.” (S.11) “I feel like I have fewer boundaries. I have the impression of being more plural compared to how I usually think, with fewer physical limits.” (S.01) “I feel both very small and infinitely vast. I am fragile, weak, and at the same time infinitely powerful” (S.07) I was here, in my body, but at the same time, because of the dissociation, I was in other bodies.” (S.13)	An expanded, less bounded or more fluid self-representation	88.2

*Note*. Occurrence (%) reflects the proportion of participants whose verbal reports included elements consistent with the described experiential pattern. As participants could report multiple experiential features, percentages do not sum to 100%.

#### Mind wandering

The verbatim reports suggest a pattern of deep absorption and attentive presence in the experience as it unfolds in the here and now (e.g. “To what I was experiencing in the moment. I was in the present of my trance, but not in the present of the room or with others” (S.01); “It wasn’t occupied with anything; it was just there, sticking to what was happening. It simply had the space to feel, to say to itself, ‘This is beautiful.’ To feel what it felt—a moment of sadness or nostalgia. It was savoring; it wasn’t jumping far away or fluttering about, but staying very close” (S.02); “I was in the lived experience of the here and now, in the present of the situation” (S.13). Interestingly, alongside this strong absorption, some participants also mentioned that their “analytical mind” remained in the background, observing the scene [e.g. “One part of me is in trance, and another part of me is reflecting, analyzing, wondering what the next movement will be, thinking ‘maybe we’ll do this’. I feel like I’m not completely letting go. My mind clings on. It’s present in the trance, but now and then, I feel it wandering” (S.04); “The presence of the mind makes small leaps as if anchoring the experience into my analytical intelligence to be able to recount it later” (S.07); “There’s maybe a small part of my rational brain telling me, ‘Maybe you should stop,’ a little fear of not being able to stop. Perhaps a small part of my brain is saying, ‘I’m in trance, but not completely disconnected from reality’” (S.09)] (see Table 3).

**Table 3:** Illustrative quotes of mind-wandering alterations during AICT versus ordinary/habitual consciousness

	Quotes—mind wandering (ID)	Synthesis	Occurrence (%)
Ordinary/habitual state	“Thinking about what I had to do afterward (after the meal).” (S.01) “My mind was back and forth. It drifted away from the food I was eating going.” (S.02) “I was doing what I was doing, but at the same time, I was thinking about all sorts of things.” (S.04) “My mind was already focused on being efficient, doing things as quickly and effectively as possible. But I could also think about other things at the same time.” (S.09)	Dispersed and Future-Oriented Attention	76.5
AICT	“To what I was experiencing in the moment. I was in the present of my trance, but not in the present of the room or with others.” (S.01) “It wasn’t occupied with anything; it was just there, sticking to what was happening. It simply had the space to feel, to say to itself, ‘This is beautiful.’ To feel what it felt—a moment of sadness or nostalgia. It was savoring; it wasn’t jumping far away or fluttering about, but staying very close.” (S.02) “I was in the lived experience of the here and now, in the present of the situation.” (S.13) “I was fully attentive—I couldn’t be elsewhere. I was catching every crumb. I wasn’t watching the film from the corner of my eye; I was 100% plugged in.” (S.03) “I was really immersed in the feeling.” (S.15)	Absorption in the Present Moment	47.0
	“One part of me is in trance, and another part of me is reflecting, analyzing, wondering what the next movement will be, thinking ‘maybe we’ll do this.’ I feel like I’m not completely letting go. My mind clings on. It’s present in the trance, but now and then, I feel it wandering.” (S.04) “The presence of the mind makes small leaps as if anchoring the experience into my analytical intelligence to be able to recount it later.” (S.07) “There’s maybe a small part of my rational brain telling me, ‘Maybe you should stop,’ a little fear of not being able to stop. Perhaps a small part of my brain is saying, ‘I’m in trance, but not completely disconnected from reality’.” (S.09) “There’s always the mental witness that watches at certain moments. It’s in the background, but never strong enough to make me stop.” (S.16) “It’s like a part of my mind was observing, saying, ‘Damn, you’re gone’, asking, ‘Is this real? Am I forcing myself to imagine this?’ And then there was a part of me taken by the emotions, in the images, in the scene. Like one part of me was living the trance and another was observing it.” (S.06)	Simultaneous Absorption and Meta-Awareness	41,2

*Note*. Occurrence (%) reflects the proportion of participants whose verbal reports included elements consistent with the described experiential pattern. As participants could report multiple experiential features, percentages do not sum to 100%.

The quantitative analyses on temporal orientation complement these findings. To examine these observations from a quantitative perspective, we performed a 2 (“AICT” vs. “ordinary state of consciousness”) repeated-measures ANOVA on the mean scores of mind wandering (i.e. past, present, future). These analyses revealed that participants reported significantly less mind wandering during the AICT compared to an ordinary state of consciousness (e.g. a meal). Specifically, during the AICT state, participants reported thinking less about past elements (*M* = 2.06, *SD* = 1.29), *F*(1, 16) = 5.71, *P* < .03, *η*^2^_p_ = .26, or future elements (*M* = 2.65, *SD* = 1.36), *F*(1, 16) = 31.9, *P* < .001, *η*^2^_p_ = .67 compared to their ordinary state of consciousness (*M* = 13.82, *SD* = 4.63 for past mind wandering and *M* = 31.47, *SD* = 5.67 for future mind wandering). Analyses also revealed that participants reported being less occupied with elements related to the present during AICT (*M* = 37.03, *SD* = 10) compared to the ordinary state of consciousness (*M* = 63.53, *SD* = 5.26), *F*(1, 16) = 8.24, *P* < .011, *η*^2^_p_ = .34 (see Fig. 1).

#### Narrative agency

We conducted the same analysis on the mean scores of narrative agency (i.e. the sense of being an agent and the sense of being an observer). The results revealed a strong significant effect of the AICT on the perception of oneself as the agent of one’s emotions and thoughts during the experience, *F*(1, 16) = 70.33, *P* < .001, *η*^2^_p_ = .81. Specifically, participants clearly expressed the sensation of no longer being the agent of their subjective experience during AICT (*M* = 12.10, *SD* = 3.30) compared to their ordinary state of consciousness in everyday life (*M* = 67.40, *SD* = 6.03). The analyses did not reveal any significant differences between AICT and the ordinary state regarding the sense of being an observer of one’s thoughts and emotions, *F*(1, 16) = 2.48, *P* = .135, *η*^2^_p_ = .13. The mean scores were, respectively, *M* = 54.71, *SD* = 9.59 during AICT, and *M* = 35.29, *SD* = 8.08 during the ordinary state (see Fig. 1).

#### Awareness of the passage of time

In the ordinary state condition, participants described their experience of time as largely aligned with real-world temporal flow. Time was perceived as continuous, predictable, and consistent with their activities [i.e. “*I think my experience of time is factual. It aligns with real time.*” (S.09); “*Time flows in a normal way*” (S.10)]. In the nonordinary state condition, AICT was associated with a profound alteration in participants’ perception of time. Some participants reported having no experience of time during their trance or described it as being “outside of time” [e.g. *“No experience of time. Timeless. I had no notion of time”* (S.13); *“There’s no experience of time. It comes afterward when you check the clock. In trance, there’s no time”* (S.14); *“It’s timeless. It doesn’t pass”* (S.05)]. Several participants also expressed difficulties estimating the speed of time, describing it instead in terms of its density and richness [e.g. *“I feel like a lot happened, but I find it hard to quantify time”* (S.11); *“It felt like a lot happened within a very short time span”* (S.10); *“I feel like it’s too fast and yet there’s an immense depth of field”* (S.08); *“It’s an experience of time that feels very full. Time feels suspended and full, but not measurable”* (S.17)] (see Table 4).

**Table 4:** Illustrative quotes of the awareness of the passage of time during AICT versus ordinary/habitual state

	Quotes—awareness of the passage of time (ID)	Synthesis	Occurrence (%)
Ordinary/habitual state	“Normal. Time passes at a normal pace.” (S.14) “I think my experience of time is factual. It aligns with real time.” (S.09) “Time flows in a normal way.” (S.10) “It goes by a little faster, but not especially slow.” (S.12) “When I’m cooking, time feels slow. But when I’m eating, it goes by fast.” (S.01)	Stable perception of time	94.1
AICT	“No experience of time. Timeless. I had no notion of time.” (S.13) “I have no idea. I don’t know whether it was long or short.” (S.09) “There’s no experience of time. It comes afterward when you check the clock. In trance, there’s no time.” (S.14) “It’s timeless. It doesn’t pass.” (S.05)	Suspension or Absence of Temporal Awareness	35,3
	“I feel like a lot happened, but I find it hard to quantify time” (S.11) “It felt like a lot happened within a very short timespan.” (S.10) “I feel like it’s too fast and yet there’s an immense depth of field.” (S.08) “It’s an experience of time that feels very full. Time feels suspended and full, but not measurable”; “Time felt hyper-condensed, extremely dense.” (S.17) “It felt long and intense. I felt like it was extremely long that I would never reach the end.” (S.16)	Temporal Distortion and Dense Subjective Time	58.9

*Note*. Occurrence (%) reflects the proportion of participants whose verbal reports included elements consistent with the described experiential pattern. As participants could report multiple experiential features, percentages do not sum to 100%.

These profound alterations in the perception of time during AICT were also reflected in quantitative evaluations. Participants indicated a significantly greater sense that time was absent during AICT (*M* = 7.00, *SD* = 2.93) compared to an ordinary state of consciousness [(*M* = 64.29, *SD* = 6.90), *F*(1, 16) = 47.96, *P* < .001, *η*^2^_p_ = .75] (see Fig. 1). However, participants also reported greater difficulty estimating the speed at which time passed during the AICT state. While all participants were able to easily estimate the speed of time during the ordinary state, 11 out of 17 participants indicated that they were unable to quantify the speed of time during AICT experience.

### Alteration of the dimensions related to the phenomenology of the minimal self

Figure 2 highlights the quantitative results for each specific alteration of the phenomenology of the minimal self.

#### Self-location

As expected, compared to the ordinary state of consciousness, the analysis of verbal reports suggests that AICT is associated with alterations in self-location. Specifically, when participants recall an ordinary daily moment (e.g. preparing a meal, having dinner with friends), their verbal reports indicate that self-location experiences were often described as centered in the upper face [e.g. “in my head” (S.01)], in the upper torso [e.g. “I feel in my body, also in my head, in the upper body” (S.07)], and for some, in their hands or stomach [e.g. “in my stomach. Under the breasts, in the stomach” (S.17)]. During AICT, the feeling of self-location becomes more diversified and complex. Some participants maintain the sensation of being located in their head (e.g. head, eyes) or in their body (e.g. solar plexus, chest, throat), while others describe the feeling of being in a different body or in a space less constrained by bodily boundaries [e.g. “I feel that I am here, but I feel located everywhere. At least, not in my head, not at all” (S.12); “I wasn’t located in a precise, closed space” (S.08); “I feel like it’s dissolved” (S.11)] (see Table 5).

Regarding the perspective from which participants perceived the scene, verbal reports suggest that, regardless of the moment recalled, most participants describe a first-person perspective from their eyes or an internal point of view [e.g. “It’s like there’s a camera here, behind the parietal. The camera is sharp. Me, the trance person, is a little blurrier” (S.08); “Completely from the inside” (S.12)] (see Table 4).

From a quantitative perspective, the 2 (AICT vs. ordinary state of consciousness) repeated-measures ANOVA on the mean scores of body identification did not reveal a significant difference between the two situations, *F*(1, 16) = 0.67, ns, *η*^2^_p_ = .04 (M = 64.11, SD = 9.32 for AICT and M = 73.10, SD = 4.33 for the ordinary state).

**Table 5:** Illustrative quotes of self-location judgements and ego/allocentric spatial frame during AICT versus ordinary/habitual consciousness

	Quotes—self-location (ID)	Synthesis	Occurrence (%)
Ordinary/habitual state	“In my head.” (S.01, S.03, S.04, S.06) “I feel in my body, also in my head, in the upper body.” (S.07) “I’m quite a lot in my head, but I also feel very much in my body. I feel pretty fully in my body.” (S12)	Self-location (head, body), embodied and clearly bounded	94
	“From my head as well.” (S.01) “I perceived it from my viewpoint, mainly through my eyes. Let’s say the camera was aligned with my eyes. So yes, I was centered.” (S.02) “Through my eyes.” (S.03) “I perceived it from myself, from the way I experienced it.” (S.11) “From me. Through my eyes, yes. Through my body, essentially.” (S.12)	First-person view from the eyes/head	100
AICT	“I was in my hands.” (S.01) “In the belly, in the chest, in the throat.” (S.09) “In the solar plexus. Also in the throat. With the sense of the plexus rising—my ‘self’ was in the breathing system from the plexus forming a diamond shape with the neck.” (S.16) “I feel that I am here, but I feel located everywhere. At least, not in my head, not at all.” (S.12) “I wasn’t located in a precise, closed space.” (S.08) “I feel like it’s dissolved.” (S.11) “I wasn’t my body, it was complicated. I felt more like I had been freed from the body, like something else was in connection.” (S.02)	Diversified and complex; diffuse or expanded forms of self-location	82.3
	“From a first-person perspective. I never see myself doing things.” (S.03) “From where I was, from my eyes. I wasn’t somewhere else. I was right where I was.” (S.04) “Yes, from my eyes. I have my head.” (S.17) “From my eyes, it’s me.” (S.9)	Egocentric view	**29.4**
	“From myself. But I feel like the view is slightly elevated, it gives me some distance”; “I see through my eyes, but what I see has no center.” (S.05) “From both inside and outside at once. It’s not like observing from above as a detached observer. I’m also the center. At first, the visualization was slightly above. It’s like having vision everywhere, not like a single drone camera, but as if it’s all at once.” (S.14) “It’s an internal gaze. I see the green, but I don’t see myself from the outside. It’s like I have eyes on the inside. I don’t see externally.” (S.11)	Mix Ego/allocentric view	41.2

*Note*. Occurrence (%) reflects the proportion of participants whose verbal reports included elements consistent with the described experiential pattern. As participants could report multiple experiential features, percentages do not sum to 100%.

Similarly, no significant difference was observed in the feeling of being at the center of the scene between AICT (M = 73.33, SD = 10.50) and ordinary life moments (M = 79.30, SD = 7.0), *F*(1, 14) = 0.22, ns, *η*^2^_p_ = .02. However, the feeling of being located in a specific point (vs. a diffuse point) was significantly affected by AICT, *F*(1, 13) = 8.48, *P* < .01, *η*^2^_p_ = .39. Participants reported feeling more diffuse during AICT (M = 50.40, SD = 10.49) compared to an ordinary state of consciousness (M = 85.70, SD = 4.19) (see Fig. 2).

#### Sense of agency

Similar to thoughts and emotions, verbal reports suggest that, compared to an ordinary state of consciousness, the sense of agency is profoundly affected during AICT. Nearly all participants mentioned the absence of control and the spontaneous and/or irresistible nature of movements or sometimes sounds [e.g. *“At the level of movements, it was irresistible”* (S.03); *“I don’t decide the movements”* (S.04); *“The gestures do what they are supposed to do”* (S.05); *“It happens spontaneously, despite myself. I allow it to happen”* (S.06); *“When the gesture is made, you feel like you did what needed to be done”* (S.05)]. Participants also occasionally noted a sense of letting go that accompanies the emergence of these motor automatisms [e.g. *“A letting go, an acceptance of what’s there”* (S.10); *“I let them unfold”* (S.17)] or an observer in the background who retains the ability to exercise control to stop the experience [e.g. *“The brain is on standby at 10%”* (S.09); *“I know that if the door opens, I will stop”* (S.14); *“It happens spontaneously, but I control the letting go”* (S.06)] (see Table 6).

**Table 6:** Illustrative quotes of sense of agency during AICT versus ordinary/habitual consciousness

	Quotes—sense of agency (ID)	Synthesis	Occurrence (%)
Ordinary/habitual state	“It’s automatic. It’s on autopilot. It’s reactive—you don’t really control it. It’s more reflexive.” (S.01) “It’s kind of autopilot, because I’m in the middle of doing something.” (S.02) “I decide something, then I do it. I organize my tasks, I organize my movements (S.10) “I have control, for sure. It’s highly controlled.” (S.03) “I’m in control.” (S.17)	Stable and voluntary agency	100
AICT	“At the level of movements, it was irresistible.” (S.03) “I don’t decide the movements.” (S.04) “The gestures do what they are supposed to do” “When the gesture is made, you feel like you did what needed to be done,” (S.05) “I didn’t do anything, it just came. I was very surprised. It started when I felt my lips trembling and lifting. It was strange and very unexpected. I didn’t decide. It was an eruption.” (S.08) “It happens spontaneously, despite myself. I allow it to happen.” (S.06) “I didn’t have the feeling that I decided anything.” (S.11)	Irrepressible and involuntary movements	**94**
	“I know that if the door opens, I will stop.” (S.14); “It happens spontaneously, but I control the letting go.” (S.06) “I had control over my body. I could have said no, but since I was OK with the trance, I was OK with everything that came with it.” (S13)	Meta-awareness and residual control	52.9

*Note*. Occurrence (%) reflects the proportion of participants whose verbal reports included elements consistent with the described experiential pattern. As participants could report multiple experiential features, percentages do not sum to 100%.

This sense of lost agency regarding body movements during AICT, compared to an ordinary state of consciousness, is statistically significant, *F*(1, 16) = 201, *P* < .001, *η*^2^_p_ = .93. When asked to quantify it, participants clearly indicated that AICT (*M* = 10.40, *SD* = 3.26), they felt they were no longer the “control tower” of their body and movements, compared to their ordinary state of consciousness (*M* = 83.80, *SD* = 4.06).

#### Self-transcendence

As expected, compared to an ordinary state of consciousness, the verbal reports suggest that AICT is closely associated with a reduction in the perceived salience of body boundaries. While participants reported feeling that their body boundaries were clearly defined during the recall of an ordinary conscious state [e.g. *“It’s very physical. It stops at the touch. It’s very limited, it’s the physical envelope in the strictest sense”* (S01); *“My body is limited to my skin”* (S07)], nearly all of them reported having more fluid, less defined, or more open body boundaries during AICT [e.g. *“I feel like some walls have been opened... there is no more barrier of the skin”* (S07); *“My boundary is diffuse, there’s something diffuse. There’s a kind of permeability, but at the same time, I still exist”* (S05); *“I don’t have the sensation of a boundary, since I don’t have it... the sensation is very strange, it’s hard to express”* (S11); *“The boundaries are no longer there. The boundaries of my body are absent”* (S14)]. Only 1 participant out of the 17 interviewed reported the sensation of very strong body boundaries during the chosen deep AICT state [i.e. *“The body is all contracted, very limited... my skin, my envelope. Everything happens inside”* (S16)] (see Table 7).

**Table 7:** Illustrative quotes of perceived body boundaries during AICT versus ordinary/habitual consciousness

	Quotes—perceived body boundaries (ID)	Synthesis	Occurrence (%)
Ordinary/habitual state	“It’s very physical. It stops at the touch. It’s very limited, it’s the physical envelope in the strictest sense.” (S.01) “My body is limited to my skin.” (S.07) “I definitely feel that I’m a limited body.” (S.12) “I clearly experience those limits, in every sense, especially since we’re sitting on the floor.”(S.16) “I clearly feel the limits. It’s quite distinct—very clear.”(S.03) “The boundaries of my body are quite precise, actually. They’re connected to my skin.” (S.06)	Clear and marked body boundaries	100
AICT	“I feel like some walls have been opened... there is no more barrier of the skin.” (S.07) “My boundary is diffuse, there’s something diffuse. There’s a kind of permeability, but at the same time, I still exist.” (S.05) “I don’t have the sensation of a boundary, since I don’t have it... the sensation is very strange, it’s hard to express.” (S.11) “The boundaries are no longer there. The boundaries of my body are absent.” (S.14) “There’s a sense of expansion.” (S.15) “I feel fuzzy boundaries. Like a ghost, soft and cottony. It’s not solid.” (S.09) “At the beginning of the trance, my boundaries were even more restricted than in a normal state of consciousness, they felt heavy, very oppressive. But as the process unfolded, they became increasingly expanded, beyond my actual physical limits.” (S.01)	Dissolution or Permeability of body boundaries	94

*Note*. Occurrence (%) reflects the proportion of participants whose verbal reports included elements consistent with the described experiential pattern. As participants could report multiple experiential features, percentages do not sum to 100%.

From a quantitative perspective, this difference in the perception of body boundaries between AICT and the ordinary state of consciousness is statistically significant, *F*(1, 16) = 36.30, *P* < .001, *η*^2^_p_ = .69. Participants feel more of a reduction in their body boundaries during AICT (*M* = 36.50, *SD* = 7.17) compared to their ordinary state of consciousness (*M* = 83.80, *SD* = 4.06). A similar pattern of results is observed for the sense of perceived duality and the feeling of oneness. Participants reported feeling less duality during AICT (*M* = 26.30, *SD* = 8.56) compared to their ordinary state of consciousness (*M* = 81.00, *SD* = 6.75), *F*(1,15) = 23.6, *P* < .001, *η*^2^_p_ = .61, and experiencing more oneness (*M* = 75.90, *SD* = 8.36 for AICT state and *M* = 28.40, *SD* = 7.40 for the ordinary state), *F*(1, 15) = 15.60, *P* < .001, *η*^2^_p_ = .51 (see Fig. 2).

#### Subjective happiness

A 2 (AICT vs. ordinary state of consciousness) repeated-measures ANOVA on the mean scores of happiness (i.e. global score and each sub-dimension: inner peace and contentment, as described by Dambrun et *al*. 2012) was conducted. The analysis revealed a significant effect of AICT on the global happiness score, *F*(1, 16) = 6.93, *P* < .02, *η*^2^_p_ = .30. Participants reported experiencing significantly more happiness during AICT (*M* = 74.2, *SD* = 6.57) compared to a selected moment in their ordinary state of consciousness (*M* = 50.50, *SD* = 6.39). The same analysis performed on each of the dimensions of the enduring authentic happiness scale revealed that it was primarily the inner peace dimension that was affected by AICT (vs. ordinary state). Specifically, participants reported feeling significantly more inner peace during AICT (*M* = 74.70, *SD* = 6.83) compared to their ordinary state of consciousness (*M* = 44.30, *SD* = 6.45), *F*(1, 16) = 8.99, *P* < .01, *η*^2^_p_ = .36. The analysis also showed a marginal difference on the contentment dimension, *F*(1, 16) = 3.91, *P* = .07, *η*^2^_p_ = .20 (*M* = 73.70, *SD* = 7.08 for the AICT state and *M* = 56.60, *SD* = 6.77 for the ordinary consciousness state).

### Relationship between sense of self during ordinary and nonordinary consciousness states and subjective happiness

We used Spearman’s rank–order correlations to explore associations between self-related variables in the AICT and ordinary states and subjective happiness scores. The results are presented in Table 8. The pattern of results suggests, tentatively and pending replication, that alterations in minimal self-related dimensions during AICT may be more consistently associated with subjective happiness than changes in the narrative self. Two correlations reached conventional levels of statistical significance: the less participants reported feeling at the center of the scene (*r* = −.59, *P* < .05) or perceiving the scene from an egocentric perspective (*r* = −.55, *P* < .05), the greater their reported subjective happiness.

**Table 8:** Spearman correlations between self-related variables during auto-induced cognitive trance (AICT) and ordinary states, time perception, and subjective happiness

	Subjective happiness	
	AICT	Ordinary state
**Narrative self**		
Mind wandering (past)	−.04	−.11
Mind wandering (future)	.05	−.49^*^
Mind wandering (present)	−.13	.41^+^
Narrative agency (agent)	−.19	.14
Narrative agency (observer)	.15	.20
**Minimal self**		
Embodiment	−.06	.30
Self-location (center)	−.55^*^	.16
Diffuse vs. localized self	−.59^*^	.36
Sense of agency (body)	−.27	.06
Bodily boundaries	−.31	.36
Self-world duality	−.34	.07
Oneness	.44^+^	−.05
**Time perception**		
Time absent/present	−.04	−.24

*Note*. ^+^*P* < .10. ^*^P < .05.

The observed correlations were of moderate magnitude and in a direction consistent with the theoretical framework for self-transcendent constructs, such as reduced bodily boundaries (*r* = −.30, *P* = .23), diminished sense of duality (*r* = −.33, *P* = .20), and heightened oneness (*r* = .43, *P* = .09). These associations did not reach statistical significance and must therefore be treated as nonsignificant findings. Given the small sample size of the present study, these effect sizes may nonetheless reflect associations worth examining in future replication studies with larger samples, but no interpretive weight should be placed on them in the context of the present investigation.

Regarding the narrative self, decreases in mind wandering, sense of agency, and increases in the sense of being an observer were not significantly associated with happiness during AICT (all *P*s > .05). In contrast, in the ordinary state of consciousness, some associations emerged. Specifically, greater engagement in future-oriented mind wandering was significantly associated with lower happiness (*r* = −.49, *P* < .05). A similar pattern was observed for present-oriented thoughts, which showed a positive association with happiness (*r* = .41, *P* = .10), however, this association did not reach statistical significance and should therefore be interpreted with caution.

## Discussion

The findings of this study suggest that Auto-Induced Cognitive Trance (AICT), compared to an ordinary state of consciousness, leads to significant alterations in the subjective experience of the self, both at the level of the narrative self and the minimal self. More specifically, with regard to the narrative self, participants in the nonordinary state condition reported a marked reduction in self-referential thought, present-moment absorption, and a sense of detachment from their habitual mental schemas, contrasting with the more structured and self-focused experience reported during the ordinary state. Several participants thus reported difficulty describing themselves using habitual trait-based terms, along with a diminished sense of narrative agency and temporal orientation. These phenomenological changes were corroborated by quantitative findings, including reduced mind-wandering (both past- and future-oriented) and a significant decrease in the perceived sense of being the agent of one’s own thoughts and emotions. The experience of time was also profoundly altered, frequently described as suspended or immeasurable.

In parallel, the findings indicate that AICT deeply affects various subjective dimensions of the minimal self, particularly in terms of self-location, agency, and perceived bodily boundaries. Compared to the ordinary state of consciousness, self-location during AICT became more fluid, diffuse, and spatially decentered. While participants in the ordinary condition typically located themselves in conventional bodily regions such as the head or upper torso, those in the AICT condition often described being situated in less defined areas (e.g. chest, throat) or even beyond the physical body. Several described themselves as “dissolved,” “everywhere,” or located in a space not confined by bodily form. AICT was also marked by a significant disruption in the sense of agency. Participants frequently reported a loss of volitional control over movements and sounds, which were described as spontaneous, automatic, or irresistibly unfolding. Instead of actively initiating movement, they portrayed themselves as passive witnesses or observers of a process that seemed to move through them rather than being generated by them. This phenomenology closely aligns with the experiential patterns described in the recent taxonomy of altered states of consciousness (Cardeña *et al*. 2025), particularly within the categories of “experiential detachment” and “altered identity states.” During AICT, individuals have reported a disruption in the usual sense of agency while maintaining a sense of bodily presence, often describing themselves as being moved by something rather than moving intentionally.

Finally, the AICT state was associated with a diminished salience of bodily boundaries and a marked shift toward self-transcendence/oneness as comparing studies focusing on meditation or other self-transcendent experience (Yaden et *al*. 2017, Dambrun et *al*. 2019, Hanley et *al*. 2020). Participants who described their bodily limits as clearly defined in the ordinary state, such as ending at the skin or at tactile thresholds, reported more porous, permeable, or entirely absent boundaries during AICT. These accounts were accompanied by a significant reduction in the sense of duality and a heightened feeling of oneness, pointing to a temporary suspension of the experiential separation between self and world. These findings raise the possibility that AICT may act as a pathway toward a transient selfless psychological functioning (Dambrun and Ricard 2011). While AICT differs from meditation in its expressiveness, embodiment, and emotional dynamics, it appears to similarly facilitate a shift away from conceptual and self-referential processing and toward a more immediate, embodied mode of awareness. The dissolution of bodily boundaries, reduced agency, and altered proprioceptive signaling observed in AICT suggest a suspension of the habitual self and world distinction, consistent with experiences of minimal selflessness. This parallels neuro-phenomenological research on meditation showing that reduced body-boundary salience and altered spatial or temporal experience are core features of self-transcendent states (Ataria *et al*. 2015). Importantly, this mode of selfless functioning does not imply disintegration but rather a fluid, decentered perspective, in which the body is no longer experienced as a rigid boundary between subject and world but as an open interface. As seen in body scan meditation (Dambrun 2016), such states allow the self to be perceived as a discontinuous and impermanent event rather than a fixed identity. AICT may thus foster not only a reorganization of self-experience but also a shift in its affective tone, enabling access to states of unity and inner peace that are often linked to self-transcendence.

In line with the SSHM, we also found that participants reported greater happiness during the AICT state than in ordinary consciousness. This effect was primarily driven by an increase in the inner peace dimension of the Authentic Happiness Scale (Dambrun et *al*. 2012). It is important to note that this well-being reflects a retrospective evaluation of the experience as a whole. From a quantitative perspective, only two correlations reached conventional levels of statistical significance, both involving spatial dimensions of the minimal self during AICT: a decreased egocentric perspective and reduced self-location at the center of the scene were associated with greater subjective happiness. The remaining correlations, although moderate in magnitude and consistent in direction with the theoretical framework, did not reach statistical significance and must therefore be treated as nonsignificant findings in the context of this pilot study. These effect sizes may nonetheless reflect associations worth examining in future replication studies with larger samples. While participants did report reductions in self-referential thought and difficulty describing themselves in stable identity terms, these narrative self changes were not significantly linked to increased positive affect. Taken together, these preliminary and largely nonsignificant correlational findings are consistent with, but do not confirm, the hypothesis that emotional benefits during AICT are primarily associated with markers of minimal selflessness rather than narrative self changes. Interestingly, this pattern is consistent with observations from meditation research, especially in practices involving deep absorption or nondual awareness (Dorjee 2016, Josipovic and Miskovic 2020, Nave *et al*. 2021), suggesting that both practices may engage shared mechanisms supporting the emergence of selfless states. Future research with larger samples is needed to test this hypothesis more rigorously and to further examine the distinction between narrative and embodied aspects of the self in relation to well-being.

Beyond these immediate associations, an important question concerns the extent to which repeated exposure to such state-level alterations of the minimal self may contribute to more enduring changes in self-experience. Two complementary theoretical frameworks provide relevant guidance here. The Pattern Theory of Selflessness (PTSL; Berkovich-Ohana et *al*. 2024) proposes that repeated experiences of self-deconstruction, characterized by reduced self-boundaries, diminished agency, and altered self-location, may progressively weaken the reification of the self as a stable, bounded entity, functioning as iterative micro-states that contribute to a gradual reorganization of the self-pattern toward greater flexibility. Converging with this account, the Mindfulness-to-Meaning Theory (MMT; Garland and Fredrickson 2019) posits that momentary states of meta-awareness and self-transcendent positive emotions can facilitate, with each iteration of practice, the development of a durable propensity toward dispositional selfless functioning and well-being. Both frameworks thus suggest that state-level self-alterations of the kind observed during AICT may represent meaningful building blocks of more enduring psychological transformation, provided that the conditions for their integration are met.

However, several important caveats must be acknowledged. First, the present study does not allow us to test this hypothesis. Our data are cross-sectional and based on retrospective reports, and therefore cannot establish whether the self-related alterations observed during AICT generalize beyond the practice context or stabilize into lasting psychological change. The mechanisms through which such transient experiences are consolidated and transferred into trait-level outcomes remain empirically unexamined in the context of AICT specifically.

Second, both the PTSL and the MMT emphasize that this progression is neither automatic nor universal. The PTSL explicitly notes that self-deconstruction experiences may be destabilizing rather than transformative when the individual’s self-pattern lacks sufficient prior flexibility (Berkovich-Ohana *et al*. 2024). This suggests that individual psychological characteristics, such as baseline psychological flexibility, prior experience with altered states, emotion regulation capacities, and the ability to integrate unusual experiences, are likely to modulate both the depth of state-level self-alterations during AICT and their potential for long-term integration. The absence of individual difference measures in the present study precludes examination of these moderating processes, and future research should address this gap explicitly.

Future longitudinal and process-based studies are therefore needed to examine whether repeated engagement in AICT practice is associated with increased self-pattern flexibility and enhanced well-being over time, and to identify the individual and contextual conditions that may facilitate or hinder the integration of these state-level changes into more durable psychological transformation.

### Limitations and future directions

This study has several potential limitations. First, the sample was composed primarily of highly educated women, many of whom were engaged in health-related professions (e.g. physicians, psychiatrists, psychologists). Additional research with larger and more diverse community samples is necessary to assess the robustness and broader applicability of these results. Relatedly, given the small sample size and the exploratory nature of the study, no a priori power analysis was conducted, and the risk of both Type I and Type II errors cannot be excluded. The large effect sizes observed for several comparisons should be interpreted cautiously, as they may be inflated in small samples. Conversely, nonsignificant results, particularly in the correlational analyses, may reflect insufficient statistical power rather than a true absence of association. Replication with larger samples is therefore necessary before firm conclusions can be drawn.

Second, to investigate the phenomenology associated with AICT compared to ordinary consciousness, we relied on participants’ retrospective recall of a significant AICT experience. Although this method has been used successfully in previous studies with experienced meditators (e.g. Droit-Volet and Dambrun 2019), it does not guarantee full or accurate retrieval of the original experience. Memory-based distortions may occur, particularly in relation to emotionally intense or temporally distant events. To address this, participants were asked to recall one of the most intense and memorable AICT experience they had ever had, increasing the likelihood of detailed and emotionally salient reports. Nevertheless, future studies could benefit from conducting semi-structured interviews immediately following AICT practice or using micro-phenomenological methods to capture more fine-grained and temporally situated descriptions (Petitmengin *et al*. 2017, 2019). Third, the control condition involved recalling a recent mealtime experience. This choice was motivated by the fact that mealtime situations are typically routine and relatively emotionally neutral, while also involving a structured temporal flow, embodied engagement, and an active narrative self. As such, they provided a meaningful and ecologically valid point of comparison with AICT across both minimal and narrative dimensions, while helping to reduce interindividual variability and offering a consistent experiential baseline. However, it should also be acknowledged that recalling a mealtime experience may involve a specific form of bodily engagement, potentially directing attention toward interoceptive or sensorimotor aspects of experience. This characteristic is not without consequence for the interpretation of the present findings. If the mealtime condition induced a relative recentering on bodily sensations and self-related concerns, it may have elevated scores on certain dimensions of the minimal and narrative self in the ordinary condition, thereby amplifying the observed differences with the AICT condition. In other words, part of the difference between conditions may not solely reflect the transformative effects of AICT but also the particular phenomenological profile elicited by the control condition itself. While this characteristic was partly aligned with our intention to include an embodied and ecologically valid control condition, it limits the extent to which the observed differences can be attributed exclusively to AICT. Future studies should therefore consider alternative or complementary control conditions, such as neutral mental imagery or resting state, to disentangle these effects more clearly.

An additional theme emerging from participants’ accounts was the experience of observing themselves from a distance, often described as a split between the part “in trance” and a background awareness monitoring the process. This dissociative-like stance was frequently linked to difficulty fully letting go and appeared to function as a psychological mechanism for maintaining control over the unfolding experience. In contrast to clinical dissociation, which typically reflects a disruption in the integration of affect, memory, and cognition (Kihlstrom 2005, Simeon and Loewenstein 2009), AICT-related experiences in this study were intentional, reversible, and often perceived as meaningful or transformative. Rather than signaling a breakdown of self-coherence, these observer-like positions may represent a transitional phase between ordinary self-structure and a more fluid, selfless mode of consciousness.

Finally, given the observed alterations of the self and their association with emotional well-being, these findings raise important questions about the potential therapeutic role of nonordinary state of consciousness practices or states (Bioy 2023, Timmermann et *al*. 2023). Our results suggest that changes in minimal self-experience, together with greater inner peace, may underlie the potential of AICT as a tool for psychological well-being.

However, these effects must be considered within the broader ecological, cultural, and socio-cognitive context in which AICT takes place. As with other altered states of consciousness, the depth and emotional tone of AICT states are likely modulated by factors such as individual intention, preparatory mindset, the facilitator’s approach, and the environmental and cultural framing of the experience (Dupuis 2021, 2022, Fort et *al*. 2025). In psychedelic research, the concept of “set and setting” emphasizes how mindset and environment critically influence experiential content and valence (Hartogsohn 2016). Similarly, Response Set Theory in hypnosis posits that expectations, motivations, and situational interpretations play a central role in the emergence and structure of subjective phenomena (Kirsch and Lynn 1998). In the present study, the fact that all participants received their training from the same institute is particularly relevant in this regard: beyond the technical components of the induction protocol, this shared training context likely conveyed common representations, expectations, and beliefs about the nature of the AICT experience. These socio-cognitively mediated expectations may have contributed to shaping the phenomenological profiles reported by participants, and cannot be fully disentangled from the intrinsic effects of the practice in the present design. Additional influences such as prior experience, group dynamics, and postexperience integration practices may further shape the meaning and impact of these altered states.

In addition, it is important to consider how specific components of the AICT practice itself may differentially contribute to the observed dimensions of subjective experience. For instance, guided imagery and verbal instructions may primarily engage narrative processes, influencing the content and structure of self-related thought. In contrast, attentional absorption, bodily focus, or sensorimotor engagement may more directly modulate minimal self-experience, including body boundaries, sense of agency, and self-location. The social and relational context may further shape both levels by influencing participants’ engagement, sense of safety, and interpretative framing. The observed alterations in both narrative and minimal self dimensions are therefore likely to reflect the combined influence of multiple interacting components rather than a single underlying mechanism.

Although the present study does not allow disentangling these different influences, future research could address these questions through more fine-grained designs, for example, by manipulating instructional framing, imagery content, or social context.

In conclusion, while most existing studies on AICT have focused on neurophysiological and experiential aspects, our findings offer a complementary perspective by clarifying how this practice can profoundly modify both the narrative and minimal self and how these changes relate to affective experience and subjective well-being. This highlights the importance of distinguishing between self-related dimensions when evaluating the psychological effects of altered states of consciousness. Future research should further explore the mechanisms and conditions under which AICT may foster self-transcendence and emotional regulation, while maintaining a critical perspective on how such transformations are shaped by contextual factors and individual mindsets.

## Supplementary Material

Supplementary_material_AICT_niag022

## Data Availability

The data that support the findings of this study are available from the corresponding author (Pierre De Oliveira) upon reasonable request.
