# Supplementary material for: From the attenuation of self-boundaries to inner peace: how nonordinary states of consciousness modulates self-experience and subjective well-being
Source: Neurosci Conscious. 2026 Aug 27;2026(1):niag022. doi: 10.1093/nc/niag022 (PMC13520798; doi:10.1093/nc/niag022)
Supplement: Supplementary file 1 [file supplementary_material_aict_niag022.docx]

**Supplementary material**

**Instructions:**

- **“AICT” Phase:**
  “We are now starting the *first* or *second* part of the interview, during which we will explore your experience of consciousness, perceptions, and sensations during a deep/intense AICT. I will ask you to think of a deep AICT experience you’ve had. To help you reconnect with that state, I invite you to take a few moments to naturally let the sensations of that deep AICT emerge. Allow yourself to gently soak in that state and reconnect with those sensations. Let me know when you feel that you are, at least partially, in that state.”
- **“Ordinary State of Consciousness” Phase:**
  “We are now starting the *first* or *second* part of the interview, during which we will explore your experience of consciousness, perceptions, and sensations during an ordinary waking state. For this, I invite you to recall *the last time you had a meal*. To help you reconnect with that state, take a few moments to naturally let the memories of that event arise: where you were, who you were with, what you ate and drank, the dishes used, and perhaps what else you were doing besides eating. Let yourself gently soak in that moment, reconnect with the sensations and feelings. Let me know when you feel that you are, at least partially, in that state.”

**Narrative Self**

For each of the following questions, they were preceded by either:
**“During this deep AICT experience,”** or **“During this event,”** depending on the condition.

- How do you perceive yourself as an individual? How would you describe yourself?
(NS – self-concept)

- What is your mind occupied with?

- To what extent is your mind occupied with thoughts about the ***past***?
(From 0% of the time to 100% of the time)

- To what extent is your mind occupied with thoughts about the ***future***?
(From 0% of the time to 100% of the time)

- To what extent is your mind occupied with thoughts about the ***present***?
(From 0% of the time to 100% of the time)

- Regarding your thoughts and emotions, to what extent do you feel like the ***agent*** of what you think and feel? (From 0% = not at all agent, i.e., on autopilot, to 100% = fully agent)

- Regarding your thoughts and emotions, to what extent do you feel like an ***observer*** of what you think and feel? (From 0% = not at all an observer to 100% = fully an observer)

**Time Perception**

- What is your experience of time during this event / during this deep AICT experience?

- How fast does time seem to pass for you?
(From 0% = very slowly to 100% = very quickly)

- To what extent would you say time is present or absent?
(From 0% = time completely absent to 100% = time fully present)

**Minimal Self**

For each of the following questions, the prompt was preceded by either:
**“During this deep AICT experience”** or **“During this event”**

- I’d like you to connect with your “self” (i.e., say to yourself internally “Me”) — where are you located? If it’s “in the body,” where exactly? If “not in the body,” do you still feel that you are this body? Where exactly?

- To what extent would you say that you are this body (or the part named)?
(From 0% = not at all, to 100% = completely)

- From where do you perceive the scene?

- Can you try to quantify how centered you feel - i.e., to what degree do you feel at the center of the scene? (From 0% = not at all, to 100% = fully centered)

- Would you say you are fully located at a single point, or instead completely diffuse?
(From 0% = completely diffuse, to 100% = fully located at a single point)

- What is your sensation of control over your body and movements? Can you describe it?

- To what extent do you feel like the control tower of your body and movements, for example? (From 0% = not at all, to 100% = fully)

- I’d like you to focus on your sensory experience of your body’s boundaries during this event Can you describe your experience?

- How would you describe the boundary between your body and everything else (your environment)? (From 0% = totally absent, to 100% = very sharply defined)

- To what extent do you feel a sense of duality between yourself and the rest?
(From 0% = no duality, to 100% = complete duality)

- To what extent do you feel at one with everything else?
(From 0% = not at all, to 100% = completely one with the rest)

**Subjective Happiness**

**During this deep AICT experience (vs. during the ordinary event), what is your level from 0 to 100% of…**

- General well-being
- Happiness
- Pleasure
- Bliss (a sense of complete happiness)
- Peace of mind
- Satisfaction
- Serenity
- Displeasure
- Beatitude (perfect happiness)
- Inner peace
- Fulfillment
- Joy
- Distress
- Inner calm
- Wholeness (a sense of full satisfaction, complete happiness, and fulfillment)
- Unhappiness
